# Deficiency of calretinin in prefrontal cortex causes behavioral deficits relevant to autism spectrum disorder in mice

**DOI:** 10.1186/s13041-025-01233-7

**Published:** 2025-07-12

**Authors:** Yaodong Zhang, Xiaotong Zhao, Chao Gao, Shengli Shi, Mengyuan Chen, Bin Guo, Shunan Hu, Daoqi Mei, Xujun Duan, Xiaona Wang

**Affiliations:** 1 Henan Children’s Hospital, Henan Key Laboratory of Children’s Genetics and Metabolic Diseases, Henan Children’s Neurodevelopment Engineering Research Center, Children’s Hospital Affiliated to Zhengzhou University, Zhengzhou Children’s Hospital, Zhengzhou, China; 2 Department of Rehabilitation, Children’s Hospital Affiliated to Zhengzhou University, Zhengzhou, China; 3 Department of Image, Children’s Hospital Affiliated to Zhengzhou University, Zhengzhou, China; 4 School of Traditional Chinese Medicine, Ningxia Medical University, Xingqing, Ningxia China; 5 Department of Neurology, Children’s Hospital Affiliated to Soochow University, Suzhou, China; 6 The Clinical Hospital of Chengdu Brain Science Institute, School of Life Science and Technology, University of Electronic Science and Technology of China, Chengdu, China

**Keywords:** Autism spectrum disorder, Calretinin, Action potential, Prefrontal cortex, Mouse model

## Abstract

Autism spectrum disorder (ASD) is a neurodevelopmental disorder characterized by core symptoms including deficits in social interaction, repetitive and stereotyped behaviors, along with higher levels of anxiety and cognitive impairments. Previous studies demonstrate pronounced reduced density of calretinin (CR)-expressing GABAergic interneurons in both ASD patients and animal models. The object of the current study was to determine the role of CR in ASD-relevant behavioral aberrations. Herein, the mRNA and protein levels of CR in the prefrontal cortex (PFC) of mouse model of ASD based on prenatal exposure to valproic acid (VPA) were determined by qRT-PCR and Western blot analysis, respectively. Moreover, the behavioral abnormalities in naive mice with CR deficiency mediated by recombinant adeno-associated virus (rAAV) were evaluated in a comprehensive testing battery including social interaction, marble burying, self-grooming, open-field, elevated plus maze and novel object recognition tests. Furthermore, the action potential changes caused by CR deficiency were examined in neurons within the PFC in naive mouse. The results show that the mRNA and protein levels of PFC CR of VPA-induced mouse ASD model were reduced. Concomitantly, mice with CR knockdown displayed ASD-like behavioral aberrations, such as social impairments, elevated stereotypes, anxiety and memory defects. Intriguingly, patch-clamp recordings revealed that CR knockdown provoked decreased neuronal excitability by increasing action potential discharge frequencies together with decreased action potential threshold and rheobase. Our findings support a notion that CR knockdown might contribute to ASD-like phenotypes, with the pathogenesis most likely stemming from increased neuronal excitability.

**Supplementary Information:**

The online version contains supplementary material available at 10.1186/s13041-025-01233-7.

## Introduction

Autism spectrum disorder (ASD) is an early neurodevelopmental syndrome associated with neurodevelopmental deficits in social communication, and stereotyped or repetitive behaviors, accompanied by anxiety and cognitive dysfunction [1, 2]. Several brain structures and functions have been confirmed to underlie ASD-like symptoms, including the prefrontal cortex (res) [3, 4]. Accumulating evidence has elegantly demonstrated that GABAergic interneurons within the PFC are strongly implicated in ASD-linked phenotypes [5–8]. The calretinin (CR)-positive cells, a calcium-binding protein, account for approximately 12–30% of the total population of GABAergic neurons [9, 10]. It was shown that the CR-containing interneurons are expressed in the rodent PFC [9]. Prior investigation showed patients with ASD had a reduced density of CR-positive interneurons in the caudate [11]. Also, recent study found decreased density of CR interneurons in medial PFC of Trio^fl/fl; Dlx5/6−CIE^ mice [12]. Of particular noteworthy, reduced neuronal excitability is thought to be critical for diverse behavioral and functional phenotypes characteristic of ASD [13–15]. Nonetheless, the mechanistic role of CR as a regulator of neuronal excitability and ASD-correlated behavioral aberrations remains to be fully clarified.

Multiple lines of evidence support that taking antiepileptic drugs, such as valproic acid (VPA) in pregnant women is related to an increased risk of developing ASD in their offsprings [16]. Converging evidence has demonstrated that prenatal exposure to VPA induces an animal model of ASD that reflects the various characteristics of individuals with ASD, including impairments in social interactions, increased levels of repetitive behavior, anxiety and cognitive defects [2, 17–19]. The overall male-to-female prevalence ratio was 3.8 of ASD among children aged 8 years [20]. Meanwhile, prenatal VPA exposure triggered male-specific changes in synaptic development, morphological development and social interaction, while female offspring manifested only marginal impairments [21]. In the current study, we explore the functional role of CR in the development of ASD-relevant phenotypes. To this end, we first examined the expression of CR in the PFC of VPA-induced male mouse model of ASD. Then, neuronal excitability in the PFC and ASD-associated behaviors of mouse with CR knockdown were investigated. These analyses revealed that downregulation of CR protein is sufficient to decrease neuronal excitability, which is liable for the evolvement of ASD-like behavioral abnormalities.

## Methods and materials

### Animals

All animal procedures were approved by the ethics committee of Zhengzhou University, following the National Institutes of Health Guidelines for the Care and Use of Laboratory Animals. Male C57BL/6J mice (aged 8–10 weeks and weighting 22–25 g were housed in a temperature and humidity-controlled (23–25 °C, 55–65%) vivarium with 12 h light cycle (lights on at 7:00 AM). Food and water were supplied ad libitum. All efforts were undertaken to minimize animal suffering and the number of animals used.

### Drug treatment

As we previously mentioned [19], male and female mice were allowed to mate overnight. Pregnancy was examined by the existence of vaginal plugs, and that day was considered as embryonic day 0.5 (E0.5). Female pregnant mice were randomly allocated to 2 groups on E12.5. Mice in experimental group were given a single intraperitoneal injection at a dose of 600 mg/kg VPA sodium salt (Sigma Aldrich, St. Louis, MO, USA; Cat# P4543) dissolved in 0.9% saline. Control group received the same volume of normal saline injection. Pups were weaned at postnatal day 21. All experiments were carried out on the male offspring. At postnatal day 35, mice were decapitated under anesthesia with isoflurane. The PFC tissue samples were rapidly dissected from the brain for the subsequent experiments.

### Quantitative real-time polymerase chain reaction (qRT-PCR)

Laser microdissection of PFC was carried out by a Laser PALM-Zeiss Microbeam system (PALM, Oberkochen, Germany) configured on an inverted Axio Observer Microscope. The expression of CR mRNA in the PFC tissues was measured using qRT-PCR analysis. In brief, total RNA was isolated with TRIZOL Reagent (Qiagen, Valencia, California, USA), followed by the reverse transcription with superScript III First-Strand Synthesis System (Invitrogen, Carlsbad, CA, USA). The transcript-derived complementary DNA was subjected to qRT-PCR analysis by the SYBRR GREEN PCR Master Mix (Thermo Fisher Scientific, Waltham, MA, USA). Glyceraldehyde 3-phosphate dehydrogenase (GAPDH) was used as the internal reference. The relative gene expression level of CR were calculated using the 2^−△△CT^ method [22]. Primer sequences used were displayed as below: CR forward 5’- CCTGCCGACCGAAGAGAATTTC-3’ and reverse 5’-GTCTGTGTCATACTTCCGCCAAG-3’; GAPDH forward 5’-TGTTGCCATCAATGACCCCTT-3’ and reverse 5’-CTCCACGACGTACTCAGCG-3’.

### Western blot

The PFC brain tissues were homogenized using ristocetin-induced platelet aggregation (RIPA) buffer containing protease and phosphatase inhibitors (Thermo Fisher Scientific). Homogenates were centrifuged at 13,000 g for 15 min at 4^◦^C. Proteins (60 µg) were separated on sodium dodecyl sulfate-polyacrylamide (SDS-PAGE) gel electrophoresis and then transferred to polyvinylidene difluoride (PVDF) membranes (Millipore, Milford, MA, USA). After blocking with 5% fat-free milk in Tris-buffered saline solution containing 0.1% Tween 20 (TBS-T) for 2 h, the membranes were incubated with the anti-rabbit CR (1:1000; Abcam, Cambridge, MA, USA; Cat# ab92341), and anti-rabbit GAPDH (1:1000; Sigma; Cat# G9545) at 4^◦^C overnight, respectively. Subsequently, the membranes were incubated with the peroxidase-conjugated anti-rabbit IgG secondary antibody (1:5000, ZSGB-Bio, Beijing, China; Cat#ZB-2301) for 2 h. Immunoreactions were exposed with enhanced chemiluminescence reagent (Pierce Biotechnology, Rockford, IL, USA) and the signal intensities were measured with luminescent image analyzer (BioRad Laboratories, Hercules, CA). The mean gray values were analyzed using Image J software program.

### Adeno-associated virus vector injection into the prefrontal cortex

The recombinant adeno-associated virus (rAAV) vectors expressing CR shRNA (rAAV-GAD67-CR-shRNA-CMV-EGFP-WPRE-hGH-ITR, 5.54 × 10^12^ vg/ml) were supplied by Shanghai Biotechnology Co., Ltd. The rAAV vectors encoding EGFP (rAAV-GAD67-CR-shRNA-CMV-EGFP, 4.83 × 10^12^ vg/ml, Fig. 2A) were applied as controls. Mice were anesthetized with isoflurane (3% for induction, 1% for maintenance) and placed in a stereotaxic frame (Xinglin Life Tech., Beijing, China). The skull was exposed and a small craniotomy was made by means of a dental drill undera stereoscopic microscope (Olympus, Co., Tokyo, Japan). Then, a volume of 2 µL rAAV-CR shRNA and rAAV-EGFP were infused bilaterally into the PFC (anterior-posterior 2.0 mm, medial-lateral ± 0.3 mm, and dorsal ventral 2.0 mm) at a slow rate (50 nL/min) using a glass micropipette (tip diameter ~ 20 μm) connected with a Nanoliter pressure micro-syringe pump and a micro controller. To prevent backflow of viral particles, the needle was remained in place for a 10 min at the end of the infusion.

Mice were randomly divided into two groups: rAAV-EGFP and rAAV-CR shRNA group. Twenty-eight days after virus injections, immunofluorescence, and Western blot analysis were used to measure the knockdown efficiency of rAAV vector interventions.

### Immunofluorescence staining

Immunochemistry was referred to our previous reports [19]. In brief, mice were deeply anesthetized, and perfusion-fixed transcardially with 4% paraformaldehyde (PFA). Brains were removed, postfixed, and transferred to 30% sucrose in 0.1 M phosphate-buffered saline (PBS) (pH 7.4) for 48 h. Coronal Sect. (40 μm) were cut on a cryostat (Leica Microsystems, Mannheim, Germany). After washing with PBS, the sections were incubated in blocking buffer containing 3% bovine serum albumin, 2% normal donkey serum and 0.3% Triton X-100 in 0.1 M PBS for 2 h at room temperature. Sections were incubated with goat anti-GFP (1:1000; Invitrogen; Cat#PA5-143588) overnight at 4^◦^C. Then, the sections were subjected to the incubation with Alexa Fluor 488-conjugated donkey anti-goat secondary antibody (1:600; Abcam, Cambridge, MA, USA; Cat#ab150129) for 2 h. After washing three times in PBS, sections were incubated with DAPI (1:1000; Invitrogen, Cat# D1306) at room temperature for 20 min and mounted onto gelatin-coated slides. Histological sections were identified using the 3DHistech MIDI Panoramic Scanner (Hungary).

### Immunohistochemistry staining for CR

Immunohistochemistry was carried out as we previously mentioned [23]. CR-Cre mice were anesthetized with pentobarbital sodium (50 mg/kg intraperitoneally), and then transcardially perfused with cold 4% paraformaldehyde. Brains were rapidly dissected and post-fixed overnight at 4 °C 4% paraformaldehyde, followed by embedding in paraffin. The brains were cut into coronal 5-µm-thick sections using a microtome (Leica Biosystems, Wetzlar, Germany). Fixed tissues were dewaxed using xylene and dehydrated with a graded series of ethanol. The tissue was exposed to 3% H_2_O_2_ for 20 min to quench endogenous tissue peroxidase and incubated in 3% normal goat serum for 30 min at room temperature. Tissue sections were incubated with the anti-rabbit CR (1:2000, Abcam, Cambridge, MA, USA; Cat# ab92341) at 4 °C overnight. After washing three times with 0.1 M phosphate-buffered saline (PBS), sections were reacted with HRP-labeled goat anti-rabbit IgG (1:1000, ZSGB-Bio, Beijing, China; Cat#ZB164117) for 2 h at room temperature. 3,3′-diaminobenzidine tetrahydrochloride hydrate (DAB) used to visualize the immune complex, stained with hematoxylin for 1 min. Images were observed and captured under an optical microscope equipped with a camera.

### Animal behavior assays

Behavioral assessments were implemented as we described previously [19]. Four weeks after rAAV vector injection, a battery of behavioral tests consist of social interaction, marble burying, self-grooming, open-field, elevated plus-maze, and novel object recognition test were performed for six consecutive days. All behavioral tests were analyzed using camera-assisted behavioral tracking software (Stoelting Co., IL, USA). All animals were handled for 3 days before behavioral tests.

### Three-chamber social interaction test

The three-chambered apparatus was 40 × 22 × 20 cm with 5 × 8 cm openings to allow exploration of each chamber. The test was carried out under dim (5 lx) intensity. A handheld lux meter was applied to check lighting in the entire compartments. Animals used as the unfamiliar strangers were age-, sex-, and strain - matched as compared with the testing mice. In the habituation phase, subject mouse was put in the empty central zone and allowed to investigate the entire chamber for 10 min. In the sociability phase, a selected novel mouse (stranger 1) was put in a wire cage (10 cm diameter) in the left chamber, and an empty wire cage (novel object) was placed in the right compartment. The location of the stranger and novel object on the left or right side was systematically changed to eliminate side preference. The testing mouse was allowed to explore the new environment freely for 10 min.

For social novelty preference test, the subject mouse was placed into the middle zone. An unfamiliar conspecific mouse (stranger 2) was put within the wire cage in the right side. The subject mouse was again allowed to freely explore each chamber for 10 min. The amount of time spent in each compartment, and time spent in sniffing or interacting with stranger mouse were both recorded. Sniffing time was as considered each instance in which a mouse’s nose touched the side chamber or the tail of the unfamiliar mouse or was oriented to it and came within 2 cm.

To evaluate sociability, we calculated a discrimination index for each mouse (time spent with novel mouse 1 - time spent with stranger object) / (total time spent with novel mouse 1 and stranger object). To measure preference for social novelty, the discrimination index was calculated (time spent with unfamiliar mouse 2 - time spent with novel mouse 1) / (total time spent with novel mouse 1 and unfamiliar mouse 2), as described previously [24, 25].

### Marble burying

Repetitive digging behaviors were evaluated with the marble burying test. A plexiglas cage (42 × 25 × 18 cm) containing 6 cm layer of chipped cedar wood bedding was used. Mice were placed individually in the cage for 15 min. Sixteen colored glass marbles (1.6 cm in diameter) were prearranged into the cage in 4 evenly spaced rows of 4 marbles each. The test was performed under low light conditions (30 lx). The number of marbles at least two-thirds buried was calculated during each test session.

### Self-grooming

Self-grooming test was performed to measure the repetitive and compulsive behaviors [26]. The mouse was introduced gently into a rectangular box (40 × 25 × 12 cm) that was covered with 2 cm of bedding, illuminated at ~ 30 lx. After 10- habituation period, the total amount of spent grooming of each mouse was recorded during a 10 min period. Grooming behaviors consist of head-washing, scratching of head and ears, and paw and leg licking. All scoring was calculated by an observer blinded to the experimental design.

### Open-field

The open-field test was carried out to address anxiety-like behavior in mice as described previously [27]. The mouse was individually placed in the center zone of an open field arena (42 × 42 × 50 cm) for a 5 min acclimatation. The illumination intensity was 30 lx. The amount of time spent in the chambers and the total distances traveled were automatically recorded during a 10 min period.

### Elevated plus maze test

The elevated plus maze test instrument composed of two open arms (30 × 5 cm) and two closed arms (30 × 15 × 5 cm) intersecting at 90° in the form of a plus connected with a middle platform. The mouse was placed in the center area of the apparatus pointing toward open arm and was allowed to explore freely for 10 min. Both the time spent in open arms and the cumulative open arm entries was calculated. Between each trial, the apparatus was cleaned with 75% ethanol and dried.

### Novel object recognition

Novel object recognition test was used to assess short-term memory changes as described previously [2] and conducted at ~ 40 lx lighting level. Mice were acclimated in the open field box (42 × 42 × 42 cm) for 10 min. During familiarization, each mouse was put into the same chamber including two identical objects of same color, size and material for 5 min. The duration of time the mouse spent in the 2 cm space around each object was measured. After a 1 h retention interval, the mouse was placed back in the box where one of the familiar objects had been replaced by a novel object and the mouse were allowed to explore for another 5 min in the test phase. The exploration time for the original and the novel object was scored. The discrimination index is calculated to be a relative measure of the distinction between the unfamiliar object and original object (t[novel]- t[familiar]) / (t[novel] + t[familiar]) × 100%.

### Brain slices electrophysiological recording

Whole-cell patch-clamp recording was performed as we described previously [28]. Mice were deeply anesthetized with isoflurane and transcardially perfused with 20 ml of ice-cold sucrose-based solution containing the following (in mM): 120 choline chloride, 2.5 KCl, 25 NaHCO_3_, 1.24 NaH_2_PO_4_, 7 MgCl_2_, 0.5 CaCl_2_, 25 glucose, 5 sodium ascorbate and 3 sodium pyruvate (300–305 mOsm/L). After decapitation, brains were transferred quickly into sucrose-based cutting solution bubbled with 95% O_2_ and 5% CO_2_ and maintained at 4 °C. Coronal brain slices (300 μm for PFC) were prepared using a vibrating microtome (5100 mz, Campden Instruments, Loughborough, United Kingdom). The EGFP-labeled neurons in the PFC layer II/III were visualized using infrared differential interference contrast microscopy (BX51WI, Nikon, Japan). The slices were continuously superfused with artificial cerebral spinal fluid (ACSF) bubbled with 95% O2 and 5% CO_2_ at a rate of 2.5 ml/min at room temperature. The ACSF contained (in mM): 124 NaCl, 2.6 KCl, 1.24 NaH_2_PO_4,_ 25 NaHCO_3_, 10 glucose, 2 MgSO_4_ and 2.5 CaCl_2_ (pH 7.4, 295–300 mOsm/L). The resistance of patch electrodes was 4–8 MΩ. For whole-cell patch-clamp, we used a potassium gluconate-based internal solution, which contained (in mM): 132 potassium gluconate, 8 NaCl, 2 KCl, 1 EGTA, 10 HEPES, 0.3 Na_3_-GTP, 2 Mg-ATP, and 0.1 CaCl_2_ (all were from Sigma) (pH 7.25–7.30, 294–297 mOsm/L). For measuring spike activity, steady-state currents were injected in 20 pA increments from 0 to 300 pA. Data were abandoned if the series resistance changed by > 20% during recording. Electrical signals were recorded with the EPC 10 amplifier and PatchMaster v2.54 (HEKA, Lambrecht, Germany). The action potentials were analyzed using Mini Analysis 6.0.3 Program (Synaptosoft Inc., USA) software.

### Statistical analysis

GraphPad Prism 26.0 (GraphPad Software, USA) was used for statistical analysis. Data are presented as the mean ± SEM. For behavioral assessment results, data were analyzed using Unpaired Student’s *t*-test or One-Way ANOVA followed by post hoc Tukey’s multiple comparison test. For the firing responses to increasing current injections, data were statistically analyzed with repeated-measures ANOVA. A difference was considered statistically significant when *p* < 0.05.

## Results

### Downregulation in mRNA and protein levels of CR

**Fig. 1:**
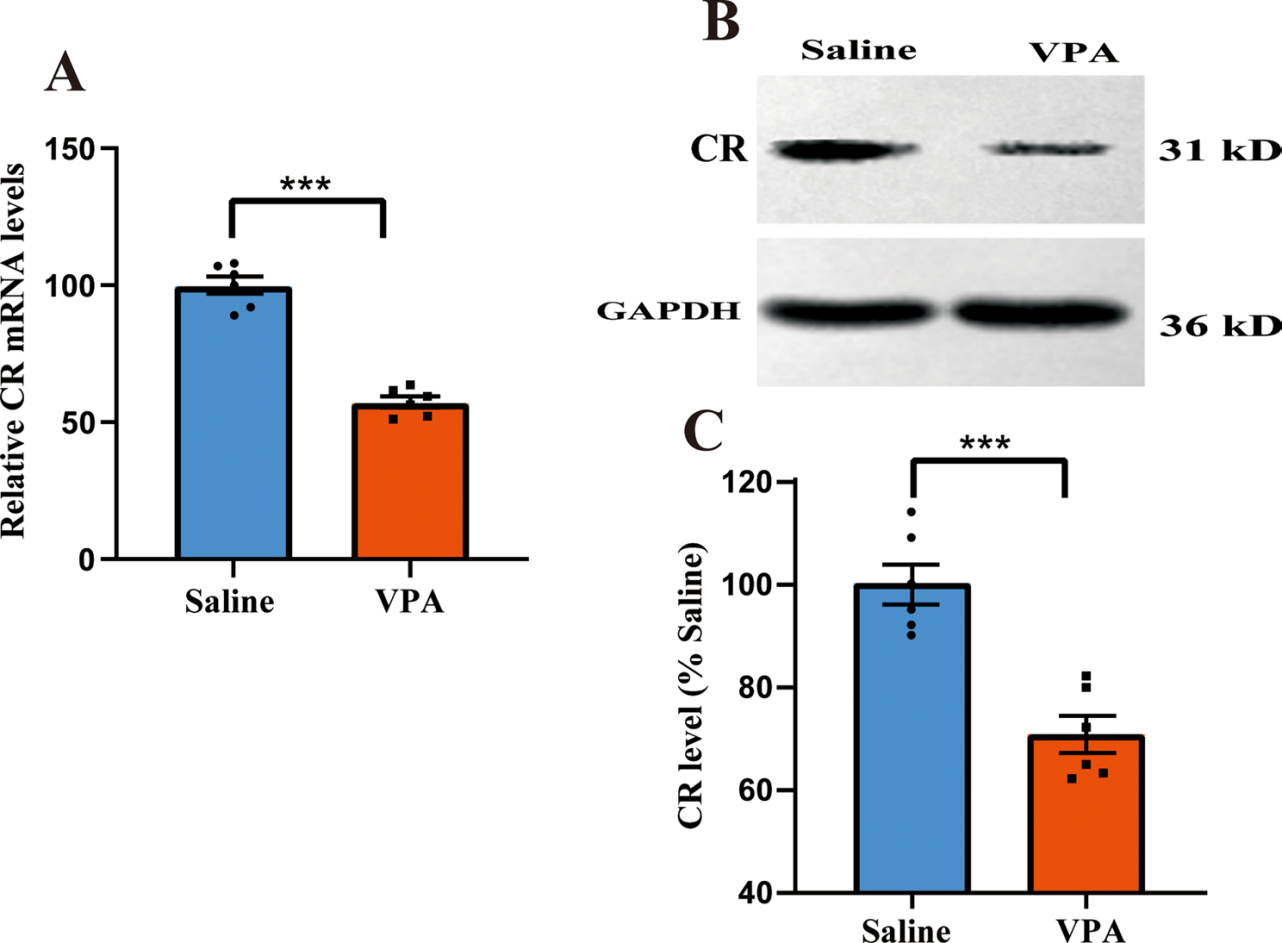
Quantitative analysis of CR expression in the prefrontal cortex (PFC) tissues of VPA-operated mice. (**A**) qRT-PCR analysis of PFC CR mRNA level from VPA-exposed animals. GAPDH was utilized as the reference gene (*n* = 6 mice per group). Student’s *t*-tests were performed. Data are presented as means ± SEM. (**B**) A representative image of the western blot. (**C**) Western blot analysis of CR protein levels in VPA-treated mice (*n* = 6 mice per group). Data are displayed as mean *±* SEM. Student’s *t*-test. GAPDH was used for the normalization of the CR protein at six separate experiments. ^***^p < 0.001 vs. saline-operated group

Firstly, the PFC CR mRNA level of VPA-induced autistic model was measured. VPA treatment remarkedly decreased CR mRNA expression in the PFC tissues as compared to that of the saline-exposed mice (*p* < 0.001, Fig. 1A). Similarly, the PFC CR protein level in VPA-induced ASD mouse model was lower than that of saline-treated animals (*p* < 0.001, Fig. 1B-C). Accordingly, CR mRNA and protein expressions both down-regulated in the PFC of VPA-treated animals.

### Confirmation of CR knockdown

**Fig. 2:**
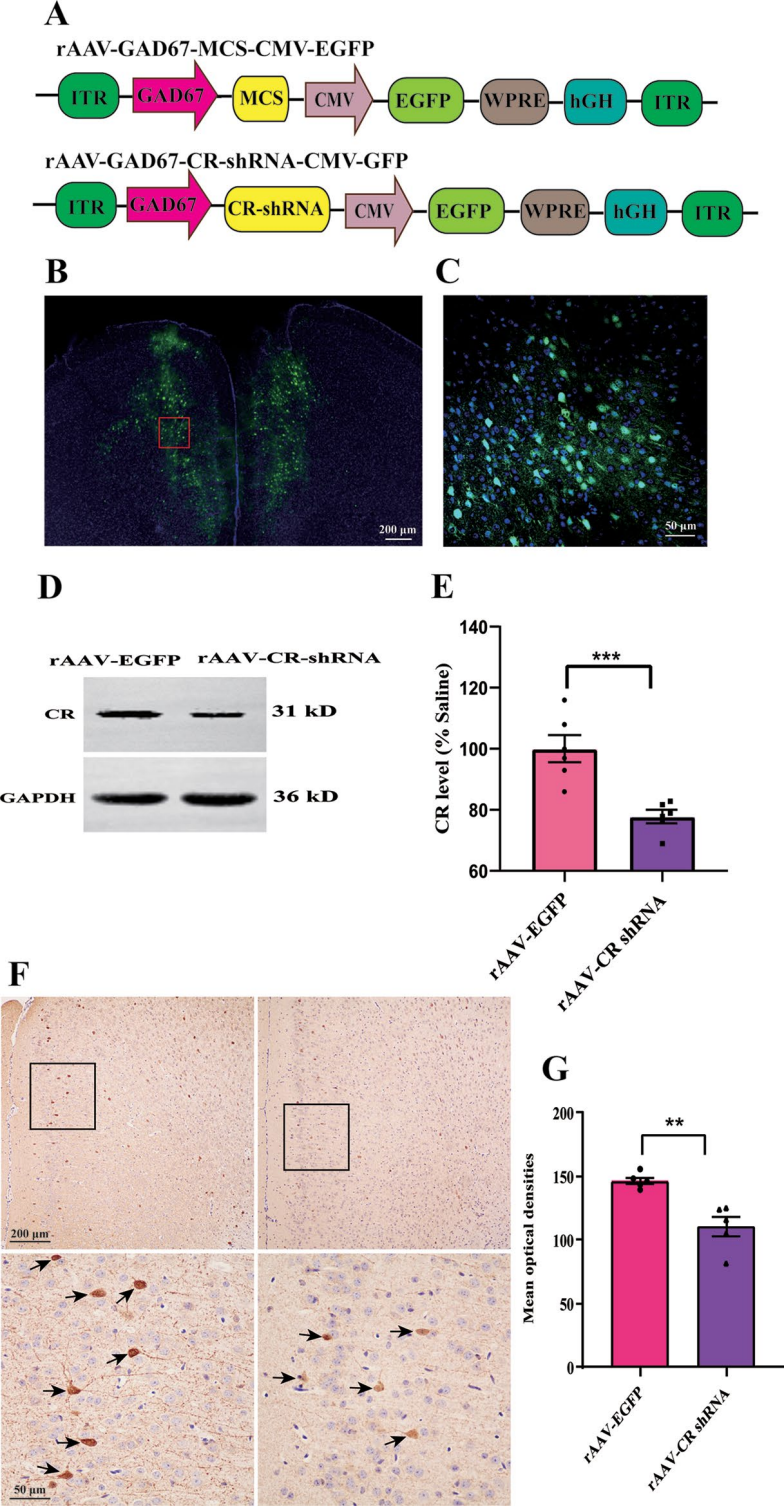
Distribution of enhanced green fluorescent protein (EGFP) and the knockdown efficiency of rAAV-CR shRNA on PFC CR expression in mice. (**A**) Viral genome maps for viruses designed to express CR and EGFP. (**B**) EGFP signals confirmed by immunofluorescence in the PFC around 21 days post injection. (**C**) The boxed area is exhibited in (**B**) at a higher magnification. (**D**-**E**) Three weeks after the injection of rAAV vectors, CR protein expression in mice with rAAV-CR shRNA intra-PFC injection (*n =* 6). (**F**) Representative images depicting CR-positive cells in sections obtained from the rAAV-EGFP- and rAAV-CR shRNA-treated animals. Upper row: Immunohistochemical detection of CR-containing cells in 10 objective lens. Bottom row: regions of PFC sketched by the black boxes in the upper row were visualized using 40 objective lens. Some CR-immunogenic neurons have been identified by arrows. (**G**) Significant expression is detected of CR interneurons in mice exposed with rAAV-CR shRNA injection (*n* = 5 each). ^**^p < 0.01, ^***^p < 0.001, compared with rAAV-EGFP-exposed animals. Student’s *t*-test. Data are presented as means *±* SEM

The knockdown efficiency of PFC rAAV-CR shRNA vectors in mice was verified using immunofluorescence and Western blot. On the 21 d after vector injection, EGFP signal in the PFC was evident, suggesting the successful sustained rAAV vector delivery (Fig. 2B-C). Animals with rAAV-CR shRNA intra-PFC injection exhibited apparently lower CR protein expression, when compared to rAAV-EGFP-exposed animals (*p <* 0.001, Fig. 2D-E). Furthermore, the expression of PFC CR interneurons in mice after rAAV-EGFP injection was decreased as compared to controls (*n* = 5, *p <* 0.01, Fig. 2F-G). These results demonstrated that our rAAV vector was able to successfully decrease PFC CR protein expression in cells in vivo.

### Deficiency of CR triggers social impairments in mice

**Fig. 3:**
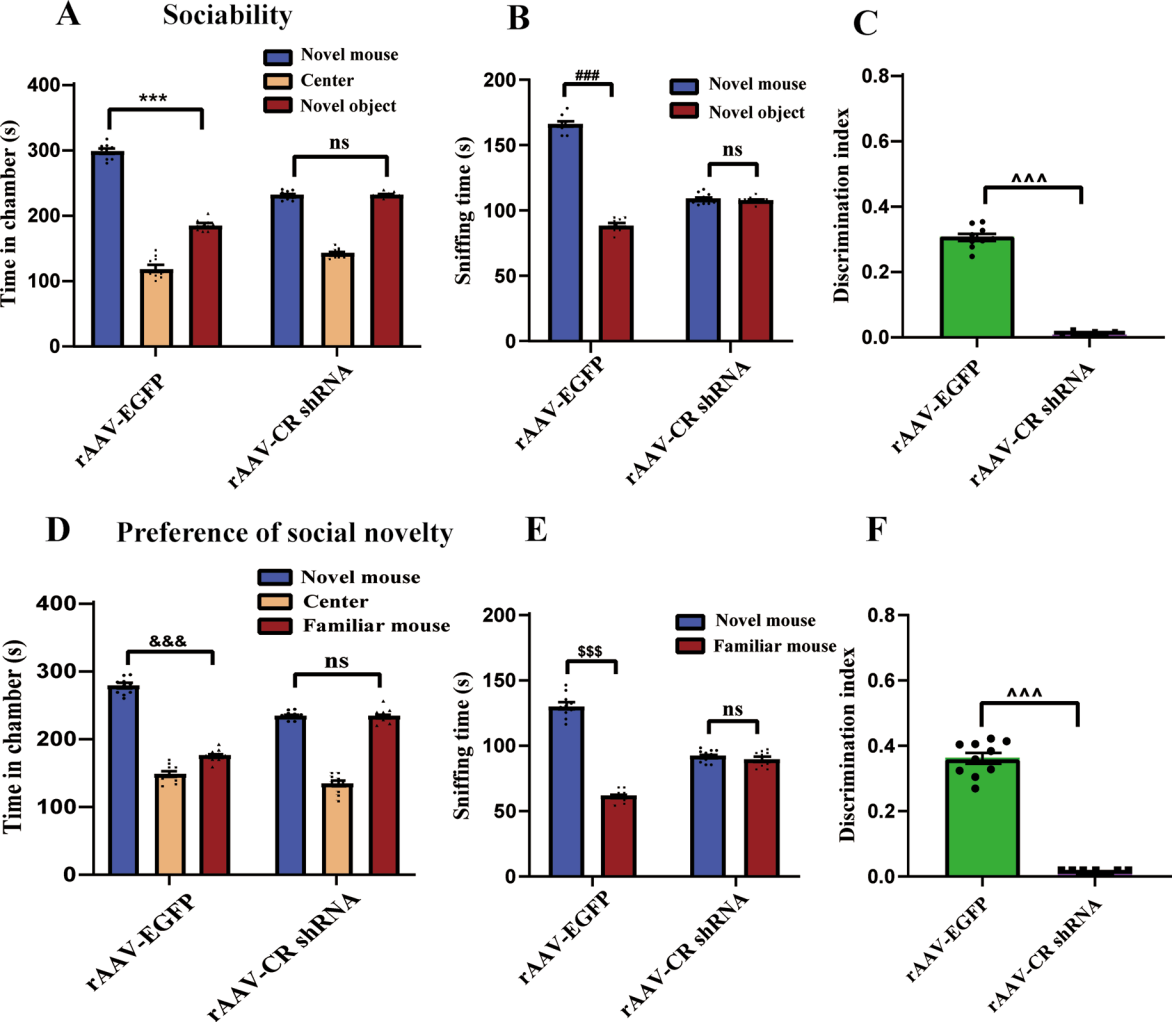
Deficiency of CR resulted in social impairments in the three-chamber test. (**A**) In three-chamber social interaction test, rAAV-EGFP-injected mice spent obviously more time in the chambers of the unfamiliar animal relative to the novel object. In contrast, rAAV-CR shRNA treatment was associated with these animals having spent equal amounts of time in the chamber containing the novel mouse and the unfamiliar object. (**B**) The rAAV-EGFP-exposed mice spent more time sniffing the novel mouse as compared to the unfamiliar object. Mice with rAAV-CR shRNA injection spent similar time sniffing with the novel mouse and the unfamiliar object. (**C**) The rAAV-CR shRNA group had an evidently lower social preference index than the control animals. *n* = 10. ^***^p < 0.001, ^###^*p <* 0.001 for novel mouse vs. novel object. ^^^^^*p <* 0.001, compared with rAAV-EGFP-treated group. “ns” indicating no statistical significance. One-way ANOVA with Tukey’s post hoc test. Student’s *t*-test. Data are means ± SEM. (**D**) In test for social novelty preferences, mice with rAAV-EGFP injection spent more time exploring the compartment containing the stranger mouse compared with familiar controls. In contrast, no considerable difference in the time spent exploring the compartment containing the novel mouse compared with the time spent exploring the familiar animal in rAAV-CR shRNA-operated mice. (**E**) The rAAV-EGFP-injected mice spent significantly more time sniffing and engaging with the novel mouse in comparison with the familiar mouse. In contrast, rAAV-CR shRNA-treated mice spent equal amounts of time sniffing the novel mouse and the familiar animal. (**F**) The rAAV-CR shRNA-administered mice had significantly a lower discrimination index than the control animals. *n* = 10. ^&&&^*p <* 0.001, ^$$$^*p <* 0.001 for novel mouse vs. familiar mouse. ^^^^^*p <* 0.001, compared with rAAV-EGFP-treated mice. “ns” indicating no statistical significance. One-way ANOVA with Tukey’s post hoc test. Student’s *t*-test. Data are presented as means ± SEM

To determine the behavioral changes with CR knockdown, the naive animal underwent sociability and social novelty preference tests. Social interactions deficits are the well-known behavioral phenotypes in individuals with ASD [29, 30]. In the sociability test, animal with rAAV-EGFP infection spent obviously longer time in chamber with an unfamiliar mouse than in empty chamber (*p <* 0.001, Fig. 3A). Nonetheless, this was not true for mice treated with rAAV-CR shRNA (*p >* 0.05, Fig. 3A). For rAAV-EGFP-injected animal, there was more time spent sniffing the unfamiliar mouse compared with sniffing with the novel object (*p <* 0.001, Fig. 3B). Animal with CR knockdown presented no particular preference, which is in consistency with the lack of interest in the unfamiliar mouse (*p >* 0.05, Fig. 3B). In comparison with rAAV-EGFP group, mice with rAAV-CR shRNA treatment showed lower discrimination index (*p <* 0.001, Fig. 3C).

Furthermore, the time spent in individual compartment in social novelty preference test was evaluated. As illustrated in Fig. 3D-F, rAAV-EGFP-exposed mice spent significantly more time in the chamber with the novel mouse than that in the compartment with the familiar animals (*p <* 0.001). No corresponding difference of time spent in exploring two compartments was detected in mouse with CR knockdown (*p >* 0.05). In addition, sniffing behaviors of mouse in the individual compartment were assessed. The rAAV-EGFP-treated mouse spent more time in sniffing and engaging with an unfamiliar mouse in comparison with a familiar animal (*p <* 0.001). Nonetheless, mouse with CR deficiency spent similar time sniffing the novel animal and the familiar mouse (*p >* 0.05). rAAV-CR shRNA-operated mice presented the obviously lower discrimination index than that of the rAAV-EGFP group (*p <* 0.001). Hence, these data were in excellent agreement with the results stem from the sociability test, suggesting that CR knockdown impaired the preference for social novelty in animal.

### CR-deficient mice display impairments in stereotyped/repetitive, anxiety and memory deficits

**Fig. 4:**
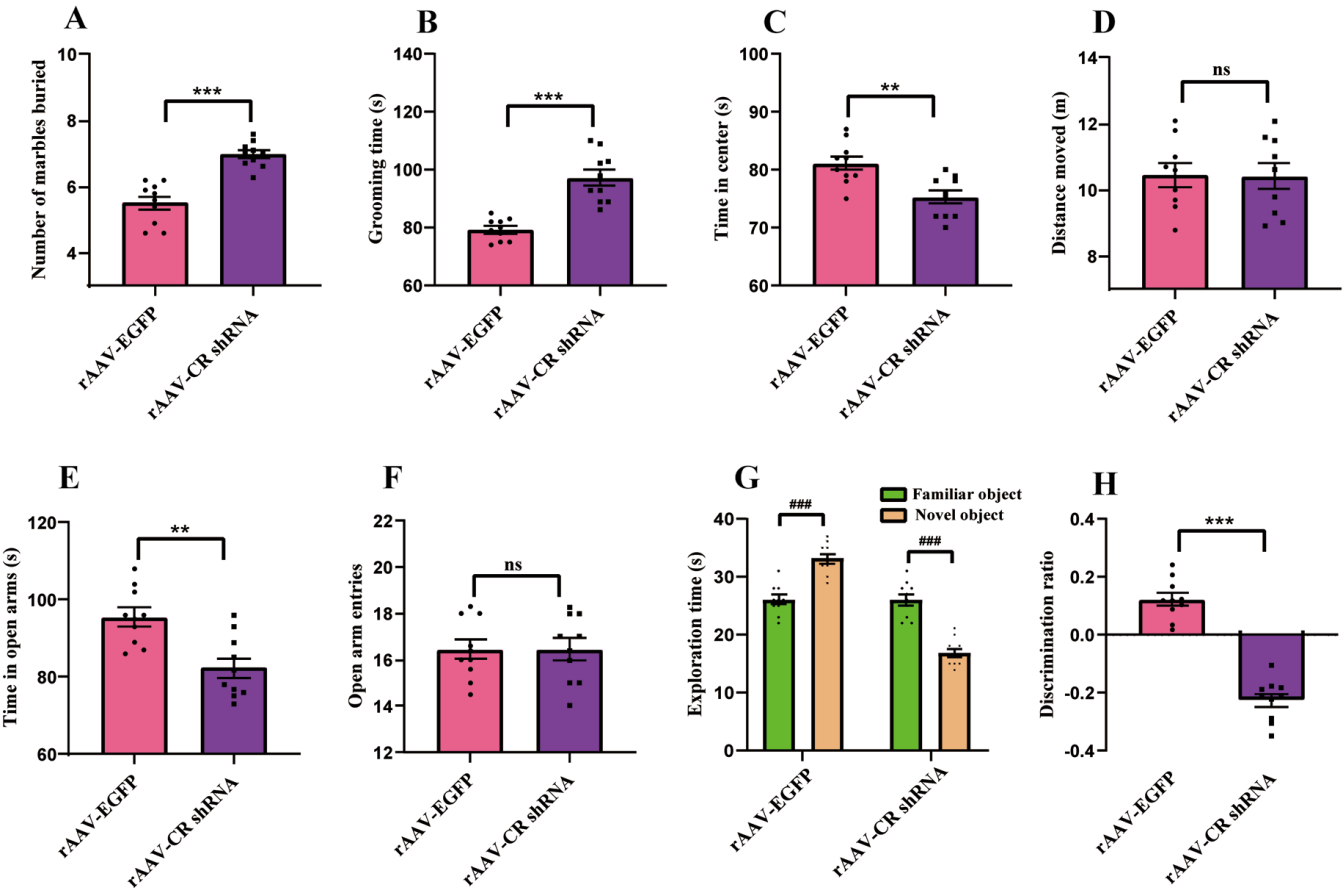
rAAV-CR shRNA injection caused stereotyped/repetitive behaviors, anxiety and memory impairments in naïve mice. (**A**) In the marble burying test, the number of marbles buried in animal with CR downregulation was increased than that of rAAV-EGFP-operated mice. (**B**) In the grooming test, mice with rAAV-CR shRNA injection spent more time in grooming relative to corresponding controls. (**C**-**D**) In the open-field test, animal with CR knockdown spent less time in the center area as compared to that of corresponding controls. No dramatical difference for the distance moved between two groups. (**E**-**F**) In the elevated plus maze test, the total amount of time the mouse with CR knockdown spent in the open arms was less than that of rAAV-EGFP-treated mice. No statistically apparent difference in open-arm entries was found between two groups. (**G**-**H**) In the novel object recognition test, rAAV-GFP-injected animals spent more time exploring the unfamiliar object as compared to the familiar one. Mice exposed with rAAV-CR injection presented significantly decreased time spent exploring the novel object in comparison with the familiar one. Animals treated with rAAV-CR shRNA exhibited decreased discrimination ratio relative to rAAV-GFP group. *n* = 10. ^***^p < 0.001, ^**^p < 0.01, compared with rAAV-GFP-treated mouse. ^###^*p <* 0.001 for novel object vs. familiar object. “ns” indicating no statistical significance. Student’s *t*-test. Data are represented as means ± SEM

The repetitive and compulsive-like behaviors can be measured by marble burying and self-grooming tests [26, 31]. In this study, we identified enhanced number of marbles buried by animal with CR knockdown relative to that of mouse with rAAV-EGFP injection (*p <* 0.001, Fig. 4A). For animal with CR downregulation, the time spent grooming was also substantially longer compared with that of corresponding controls (*p <* 0.001, Fig. 4B).

In order to identify whether CR knockdown lead to anxiety-associated behavior in mouse, open-field and elevated plus maze tests were performed. As seen in Fig. 4C-F, animal with CR knockdown spent less time in the center of open field in comparison with that of the rAAV-EGFP group (*p <* 0.01), while no apparent difference for the distance moved between two groups was observed (*p >* 0.05). Furthermore, in the elevated plus maze test, treatment with rAAV-CR shRNA was associated with reduced time spent in open arms relative to rAAV-EGFP-exposed mice (*p* < 0.01). The number of open-arm entries was comparable between two groups (*p >* 0.05).

ASD is characterized with early onset of memory alterations [32, 33]. In the novel object recognition test, rAAV-EGFP-operated animal spent longer time in exploring the novel object relative to the familiar object (*p <* 0.001, Fig. 4G). However, mouse with CR knockdown spent less time exploring the unfamiliar object in comparison with the familiar one (*p <* 0.001, Fig. 4G). Remarkably, animal with CR knockdown showed reduced discrimination ratio comparing with that of control group (*p <* 0.001, Fig. 4H). Together, the discussed results illustrate that PFC CR knockdown resulted in core ASD-associated phenotypes such as stereotypical repetition, anxiety and memory impairments.

### CR knockdown decreases neuronal excitability

**Fig. 5:**
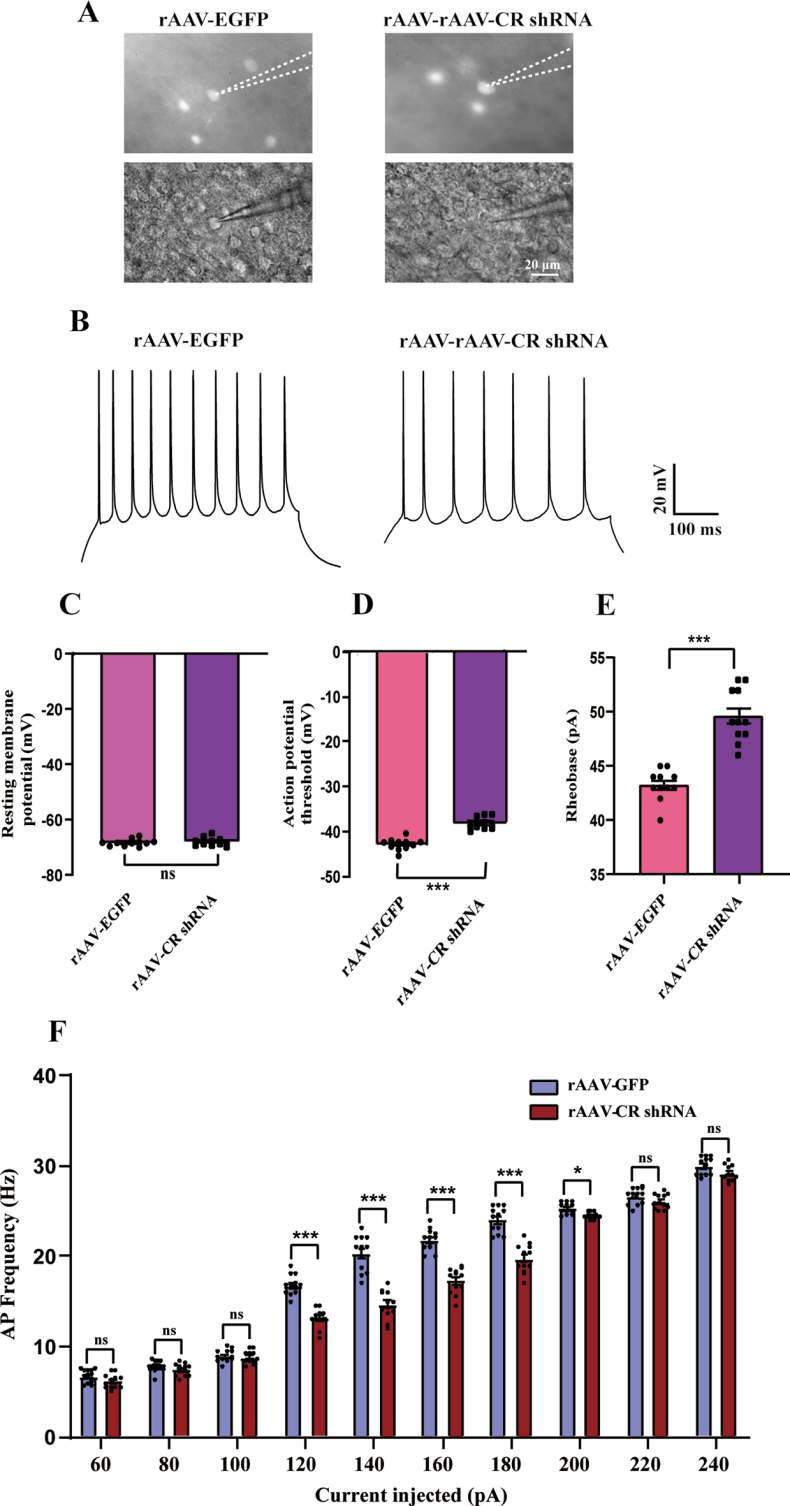
In vitro whole-cell patch recordings showing the impact of CR knockdown on neuronal excitability in the PFC slices. (**A**) Fluorescent (EGFP, upper panel) and infrared differential interference contrast (DIC, bottom panel) image showing EGFP-labeled cell patched in the PFC from rAAV-EGFP and rAAV-CR shRNA mice. (**B**) Representative spiking pattern of EGFP-containing neurons traces in response to 200 pA current injection. (**C**-**E**) Quantitative comparisons of the resting membrane potential, action potential threshold, and rheobase current. (**F**) Evoked spike rates vs. current magnitudes in the PFC between two groups, suggesting a significant increase in mean firing frequency in rAAV-CR shRNA-treated mice compared to rAAV-EGFP group (*n* = 11 cells for rAAV-GFP from 5 animals and 11 cells for rAAV-CR shRNA 5 animals). ^***^p < 0.001, ^**^p < 0.01, “ns” indicating no statistical significance; repeated measures ANOVA. Data are means ± SEM

The reduced cortical excitation is linked to key behavioral traits of ASD [13, 15]. Patch-clamp recordings was conducted to examine the influence of PFC CR deficiency on excitability in EGFP-positive cells. Fluorescent and infrared differential interference contrast images of representative CR interneurons are displayed in Fig. 5A. Representative traces of action potential using the 200 pA depolarizing current injection are manifested in Fig. 5B. We explored the passive and ace membrane properties in the current-clamp mode. The resting membrane potential of evoked action potential were comparable between two groups (*p* >0.05, Fig. 5C). Nevertheless, the action potential threshold was evidently increased in rAAV-CR shRNA-treated mice (*p* < 0.001, Fig. 5D). Similar results were also detected for rheobase (*p* < 0.001, Fig. 5E). These analyses showed a significant decrease in action potential discharge frequency in slices of EGFP-expressing cells from rAAV-CR shRNA mice compared to control group (depolarizing steps ranging from 120 to180 pA, *p* < 0.001; 200 pA depolarizing steps, *p* < 0.05; Fig. 5F). Consequently, these data were consistent with the animal models in ASD wherein CR down-expression can reduce neuronal excitability.

## Discussion

Our findings were the first to reveal CR knockdown in the PFC of VPA-injected animals at both the mRNA and protein levels. Also, we found that downregulation of CR was sufficient to result in typical ASD-associated phenotypes in animals, as manifested by reduced social interactions and repetitive behaviors, anxiety-like traits, and memory deficits. Furthermore, CR knockdown was able to decrease neuronal excitability in the PFC of naive mouse. The current data are very interesting because we demonstrate that CR hypofunction was implicated in ASD-like behavioral abnormalities, in part due to impaired excitability.

The remarkablely reduced density of CR-positive interneurons has been reported in the caudate of individuals with ASD [11] and medial PFC in Trio^fl/fl; Dlx5/6−CIE^ mice [12], indicating that dysfunction of this essential CR interneurons might contribute to the functional impairments in ASD. Herein, Western blot and qRT-PCR analysis revealed decreased CR expression in the PFC of VPA-induced ASD mouse model. We therefore raised the hypothesis that CR knockdown was responsible for the ASD-relevant behavioral deficits. To this end, we explored whether PFC CR downregulation is involved in the presence of ASD-like characteristics in naive mouse. The key symptoms of ASD encompass defects in social and communication [34]. An overwhelming body of evidence revealed impaired sociability and preference for social novelty in the mouse model of ASD caused by prenatal exposure to VPA [35–37]. Consequently, our investigation demonstrated that mice with CR knockdown in the PFC exhibited ASD-associated symptoms, including defects in sociability and preference for social novelty.

Repetitive and stereotyped behaviors are widely accepted as core diagnostic features of ASD [38]. A great deal of data reveal that in utero VPA-treated mice displayed enhanced number of marbles buried in marble burying test [35] and longer time spent grooming during self-grooming test [39]. Noticeably, we found that CR-deficient mice displayed repetitive and stereotyped behaviors, as evidenced by marble burying and self-grooming tests. These findings implied that CR downregulation might contribute to the ASD-related behavioral abnormalities, such as stereotype and repetition. Both genetic and environmental factors indicated that PFC is implicated in repetitive behaviors [40–42]. The application of GABA-enhancing drugs mitigate self-grooming behaviors in rodents [41]. Using single-nucleus RNA sequencing, Lv et al. demonstrated that the overactivation of protein kinase C (PKC) in Neuroligin 1-deficient mice contributes to excessive repetitive behaviors. Additional experiments should be conducted to elucidate whether overactivation of PKC can lead to repetitive behaviors in animal with CR knockdown.

It is well known that individuals with ASD are at enhanced risk for developing anxiety [43]. Kruse et al. found that rats administrated to sucrose during youth presented an upregulated expression of CR-immunoreactivity in the medial PFC related to anxiety in adulthood [44]. The CR gene downregulation in the amygdala of mice lacking type 2 deiodinase could affect the GABAergic transmission, which led to enhance anxiety [45]. Moreover, experimental data showed that photoactivation of CR-containing interneurons in zona incerta produced anxiolysis in mice [46]. Chen et al. reported that VPA increased anxiety levels in mice [47]. Our work revealed that animals with CR downregulation in the PFC trigger higher levels of anxiety. In line with prior results, the present study implicates that CR deficiency enhances susceptibility to develop anxiety-like phenotypes.

It is generally recognized that memory alterations memory dysfunctions were present in children with ASD and animals [48]. Growing evidence suggests that prenatal VPA exposure causes defects in short-term recognition memory [49, 50]. There was a marked reduction in the proportion of CR-immunoreactive neurons in individuals with Down syndrome [51]. Mátyás et al. stated that a numerical decrease of hippocampal CR-containing GABAergic neurons were responsible for the loss of the learning and memory functions in the pilocarpine model of temporal lobe epilepsy [52]. In conjunction with these findings, we propose that PFC CR knockdown was capable to induce memory deficits in animals.

The mechanisms whereby CR downregulation results in core ASD-relevant symptoms in naive animals remain to be clarified. Currently, it is noted that these harmful effects might be associated with the augmentation of in neuronal excitability caused by CR knockdown. Significant efforts have been made to unravel that CR has a powerful influence on neuronal excitability [10, 53, 54]. We showed that CR downregulation increased the threshold voltage for action potential firing and rheobase of CR neurons in the PFC slices by whole-cell patch-clamp recordings. Also, CR deficiency causes decreased action potential discharge frequencies from naive mice. These changes reduced CR-expressing neurons’ excitability. In conjunction with our findings, Cao et al., found that neuroligin 3 R451C knockin mice with social deficits exhibit the impaired intrinsic excitability of parvalbumin (PV) interneurons in the mPFC [14]. Brown et al., reported that increased thresholds of action potentials were detected in cerebellar Purkinje neurons of Tsc1 deletion mouse models of ASD [55]. Noticeably, the threshold and rheobase for action potential generation were enhanced and the frequency of action potentials within trains was reduced of somatostatin- and PV-expressing cortical interneurons from mice with haploinsufficiency in the gene encoding the NaV1.1 sodium channel, which is associated with social abnormalities [56, 57]. Further researches are required to more specifically target whether CR deficiency culminates in impaired excitability owing to deficiency in NaV1.1 sodium channel.

Our observations are were in harmony with previous reports. An increasing number of studies have shown that cortical PV-containing interneurons are hypoactive in genetic models of ASD displaying social deficits, including those strongly associated with mutations in Shank3, Fmr1 and Mecp2 [58]. Another compelling evidence suggests that PV interneurons hypofunction is a principal driver of social deficits in ASD [59]. Congruent with these findings, decreased cortical excitability is critical for multiple behavioral and functional phenotypes of ASD [13–15]. Indeed, when CR in the PFC was deficient in the naive mouse, we concluded that it induced ASD-related behavioral impairments owing to decrease in neuronal excitability.

It was demonstrated that Mef2c heterozygosity in developing GABAergic cells triggered reductions in PV cell intrinsic excitability and produced deficits in social preference [15]. Also, there is ample evidence that loss of UBE3A resulted in reduced excitability of layer 5 fast spiking interneurons in the deletion of ubiquitin protein ligase E3A mouse model of Angelman syndrome [60]. Routh et al. considered that PFC layer 2/3 pyramidal neurons display higher gain of somatic excitability in Fmr1-/y mice, responding with a higher number of action potentials for a given stimulus [61]. Although excitatory/inhibitory synaptic imbalance is evident in several ASD models, it is not always associated with enhancement in the firing rates of excitatory pyramidal cells. Thus, it has been accepted that changes in feed-forward inhibition may represent a compensatory rebalancing to reduce excitation [58].

Summing up, the present data indicate decreased CR expression in the PFC of VPA-induced rodent model of ASD. Recombinant adeno-associated virus-mediated RNA interference of CR in native mice is sufficient to decrease neuronal excitability, and thereby triggers core ASD-related phenotypes. Our findings provide novel insight for the ASD research community and can be further explored for a role of CR as a potential target through combination of genetic and pharmacological manipulations.

## Electronic supplementary material

Below is the link to the electronic supplementary material.

Supplementary Material 1

## Data Availability

No datasets were generated or analysed during the current study.
