# Supplementary material for: Deficiency of calretinin in prefrontal cortex causes behavioral deficits relevant to autism spectrum disorder in mice
Source: Mol Brain. 2025 Jul 12;18:61. doi: 10.1186/s13041-025-01233-7 (PMC12255998; doi:10.1186/s13041-025-01233-7)
Supplement: Supplementary file 1 — Supplementary Material 1 [file 13041_2025_1233_MOESM1_ESM.pdf]

Full uncropped Gels and Blots images

Saline VPA

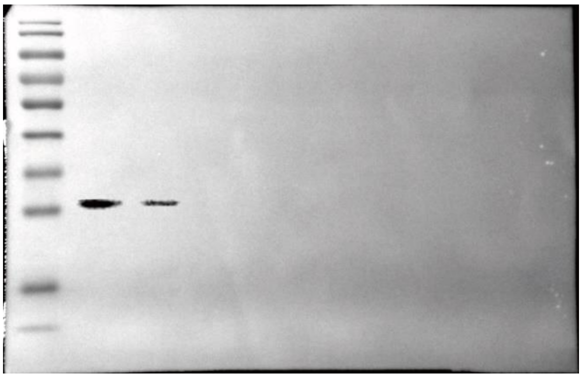

CR

Saline VPA

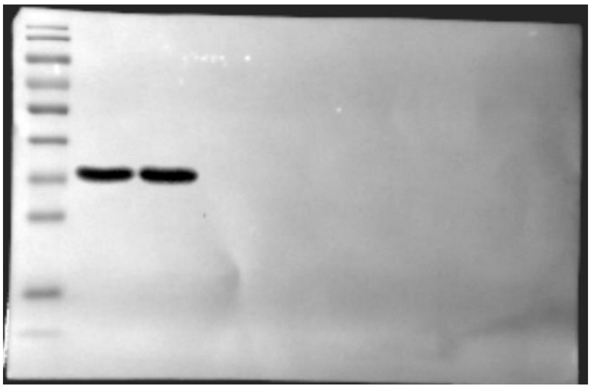

GAPDH

rAAV-EGFP rAAV-CR-shRNA

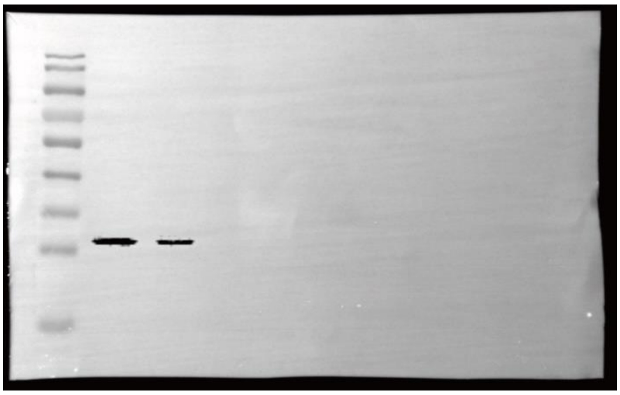

CR

rAAV-EGFP rAAV-CR-shRNA

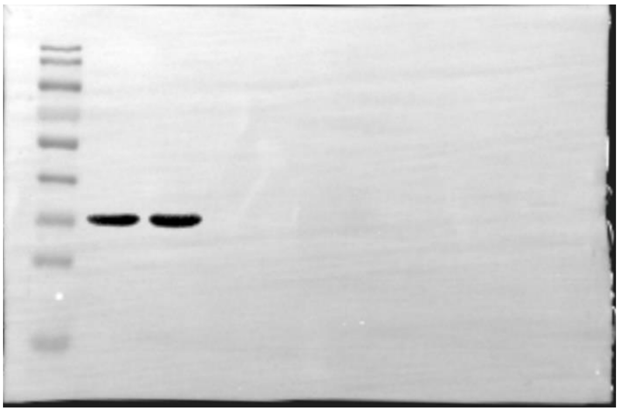

GAPDH
